# Understanding the Process of Fibrosis in Duchenne Muscular Dystrophy

**DOI:** 10.1155/2014/965631

**Published:** 2014-05-04

**Authors:** Yacine Kharraz, Joana Guerra, Patrizia Pessina, Antonio L. Serrano, Pura Muñoz-Cánoves

**Affiliations:** Cell Biology Group, Department of Experimental and Health Sciences, Pompeu Fabra University (UPF), CIBER on Neurodegenerative diseases (CIBERNED), Institució Catalana de Recerca i Estudis Avançats (ICREA), Doctor Aiguader 83, 08003 Barcelona, Spain

## Abstract

Fibrosis is the aberrant deposition of extracellular matrix (ECM) components during tissue healing leading to loss of its architecture and function. Fibrotic diseases are often associated with chronic pathologies and occur in a large variety of vital organs and tissues, including skeletal muscle. In human muscle, fibrosis is most readily associated with the severe muscle wasting disorder Duchenne muscular dystrophy (DMD), caused by loss of dystrophin gene function. In DMD, skeletal muscle degenerates and is infiltrated by inflammatory cells and the functions of the muscle stem cells (satellite cells) become impeded and fibrogenic cells hyperproliferate and are overactivated, leading to the substitution of skeletal muscle with nonfunctional fibrotic tissue. Here, we review new developments in our understanding of the mechanisms leading to fibrosis in DMD and several recent advances towards reverting it, as potential treatments to attenuate disease progression.

## 1. Introduction

Duchenne muscular dystrophy (DMD) is a fatal, X-linked recessive disorder characterized by a progressive loss of muscle mass and function [1]. DMD has a prevalence of approximately 1 in 3,500 live male births and is caused by mutations in the dystrophin gene that precludes the synthesis of a full-length and/or fully functional protein. Dystrophin itself is a large structural protein that stabilizes the sarcolemma of muscle fibers. In its absence, fibers become vulnerable to contraction-induced damage and undergo cycles of necrosis and repair until the muscle mass becomes replaced by fat and fibrous tissue. Affected boys become confined to a wheelchair and normally live until the late teens or early twenties. There is currently no cure and patients only receive palliative care. Consequently, there has been a considerable and sustained effort to uncover the mechanisms of disease and develop new treatment possibilities [2, 3]. Attempts to provide a primary treatment of DMD include viral replacement therapy, plasmid-mediated nonviral expression, stem cell transplantation, antisense oligonucleotide-induced exon skipping, and nonsense mutation suppression by drugs, amongst others, although they remain unsuccessful [4–7].

Recently, however, attention has also begun to focus on understanding and modifying the pathological background of the disease, it is now well established that many of the pathological features of DMD are not only caused by the lack of dystrophin and/or the failure of muscle stem cells (also called satellite cells) but are also due to the complex interactions of these cells with the surrounding environment. Changes in this environment can delay muscle repair and regeneration and enhance inflammation, leading to disease exacerbation and fibrosis development. Important contributors to DMD pathogenesis, and potential obstacles or targets for achieving better therapeutic outcomes, include the inflammatory components of the damaged and regenerating muscle and the auxiliary cell mediators such as fibroblasts that support satellite and inflammatory cells, as well as the milieu of soluble factors produced by them. In particular, recent studies have highlighted the importance of all these cells and factors in the development of not only fibrosis arising during aberrant regeneration and DMD progression, but in other diseases such as diabetes and in aging (reviewed in [8]).

Fibrosis is defined as the excessive or unregulated deposition of extracellular matrix (ECM) components and is a particular hallmark of DMD and abnormal repair processes in several other tissues upon injury including liver, lung, kidney, and pancreas. Controlled deposition of ECM components during growth and repair is critical for providing a scaffold to build and structure new tissue, but alterations in the timing, the intensity, and/or the components of this process can lead to excessive ECM deposition (fibrosis) and loss of tissue function. Fibrosis has a double negative consequence for the potential treatment of DMD in that it not only alters muscle function, but also reduces the amount of target muscle available for therapy and repair. Therefore, a better understanding of the components and processes leading to the development of fibrosis is important for our ability to improve muscle repair, treat DMD, and potentially restore muscle function. Although this review focuses on DMD, there is evidence from other myopathies that a dysregulated and/or disordered ECM may also contribute to disease progression [9, 10].

A review of all of the contributing factors that lead to fibrosis is beyond the scope of this report, although several recent reviews are available [11, 12]. Instead we focus here on some of the recent developments that reveal new or unexpected roles of some of these cell types and molecular effectors of skeletal muscle fibrosis, giving particular emphasis on fibrosis in DMD patients, as well as in animal models such as the commonly used dystrophic mdx mouse. In particular we highlight some new developments in the understanding of the TGF*β* signaling pathway, perhaps the most critical effector of fibrosis, and in particular its interaction with connective tissue growth factor (CTGF) and the renin angiotensin system (RAS). We also review recent efforts to lineage trace cellular sources of ECM production and assess the novel fibrogenic role assigned to other nonsatellite cell types that have been identified in muscle. Finally, we will also describe some of the recent progress in the development and characterization of animal models for the study of fibrosis* in vivo* and some potential therapeutic approaches to combat and diminish fibrosis in DMD.

## 2. Growth Factors in Fibrosis Development

### 2.1. The TGF*β* Signaling Pathway

One of the most potent profibrogenic factors described* in vivo* is transforming growth factor-beta (TGF*β*) as reviewed in [13]. TGF*β* is initially generated as a latent precursor of one of three isoforms TGF*β*1, TGF*β*2, and TGF*β*3 [14]. Latent TGF*β* is stored in the ECM where it is activated upon tissue damage or specific growth signals (reviewed in [15]). Activated TGF*β* binds to a heterodimeric complex consisting of one TGF*β* type I receptor molecule, also called activin linked kinase 5 (ALK5), and one TGF*β* type II receptor. Importantly, TGF*β* is expressed in regenerating muscle after injury, as well as in the dystrophic muscle of DMD patients and mdx mice [14, 16, 17], where it stimulates fibroblasts to produce ECM proteins like collagen and fibronectin. In addition, TGF*β* and other profibrogenic polypeptides can be produced by infiltrating immune, inflammatory, mesenchymal, and tissue-specific cells (reviewed in [18]).

**Figure 1:**
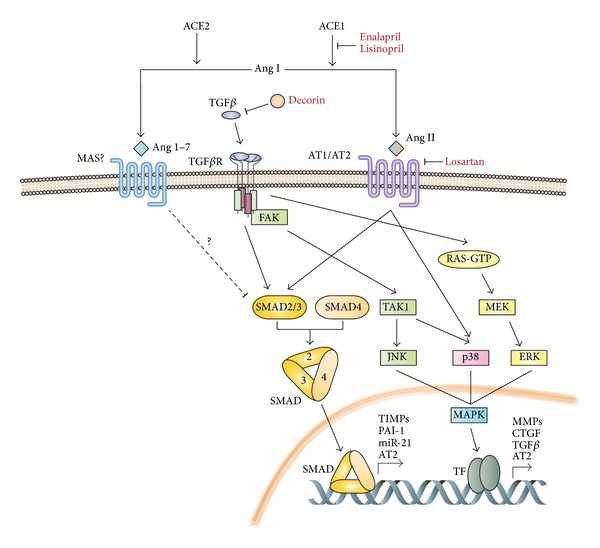
Crosstalk between TGF*β* signaling and the renin-angiotensin system in fibrosis. TGF*β* can signal via its canonical pathway, involving Smad proteins, or through several alternative pathways such as the p38 MAPK signaling or the RAS/ERK MAPK signaling pathways. Both canonical and alternative pathways lead to expression of molecules implicated in fibrosis such as CTGF or PAI-1. Similarly, Ang II signals through AT1 or AT2 and can also activate Smad proteins and the p38 MAPK signaling pathway, leading to increased expression of profibrotic genes. Ang 1–7 has an opposite effect, inhibiting the canonical TGF*β* pathway. Antifibrotic molecules inhibiting RAS or the TGF*β* signaling are indicated in red.

When TGF*β* is liberated it can signal via the canonical TGF*β* pathway (see below) or through several alternative pathways (Figure 1). Importantly, changes in the level of signal transduction via these different pathways have been shown to modulate fibrotic effects and therefore their various signaling mediators are potential targets for antifibrotic therapies. In normal fibroblasts the canonical pathway passes through ALK5 which phosphorylates transcription factors Smad2 and 3. These signal transducers then bind to Smad4 to form a complex that is translocated to the nucleus to activate transcription of profibrotic genes (reviewed in [19, 20]). Alternatively, TGF*β* may also signal via additional intracellular transducers such as the Ras/MEK/ERK pathway, the p38 MAPK pathway, the c-abl pathway, and JNK as additional intracellular mediators [21]. Signaling via these alternative pathways is able to modify gene expression in a promoter-selective fashion. ERK, for example, is normally required for collagen type I expression, whereas other signaling molecules like FAK, JNK, and TAK1 are required for divergent processes such as ECM contraction and myofibroblast differentiation [22].

Recently, several indirect interactions between TGF*β* signaling and other pathways have been reported. For instance, decreased insulin-like growth factor (IGF) signaling in IGF-1R(+/−) heterozygous mice deleted for the IGF-1 receptor in skeletal muscle using a muscle specific MyoD-Cre driver resulting in impaired regeneration, depressed expression of MyoD and myogenin, and increased expression of TGF*β*1, *α*-SMA, and collagen I and fibrosis [23]. Further mechanistic studies showed that in myoblasts, IGF-1 treatment could inhibit TGF*β*1-stimulated Smad3 phosphorylation and increase phosphorylated-AKT- (P-AKT-) Smad3 interactions, thus impeding nuclear translocation of Smad3 and thereby reducing the expression of fibrotic genes. Conversely, a reduced amount of IGF-1R diminished the levels of P-AKT, allowing dissociation and nuclear translocation of Smad3 and enhancement of the TGF*β*1 signaling pathway and fibrosis [23].

TGF*β* can also decrease the production of enzymes that degrade the ECM, while simultaneously increasing production of proteins like tissue inhibitors of metalloproteinases (TIMPs) and plasminogen activator inhibitor type-1 (PAI-1, see below) that inhibit ECM-degrading enzymes. Consistent with this, injection of recombinant TGF*β* into skeletal muscle* in vivo* has two effects. Firstly, it stimulates TGF*β* expression in myogenic cells (among other cells) in an autocrine fashion and secondly, it induces connective tissue production in the area of the injection (reviewed in [24]). The same study has shown that C2C12 myogenic cells overexpressing TGF*β* can differentiate into myofibroblastic cells after intramuscular transplantation [25]. This process can be inhibited by the small leucine-rich proteoglycan decorin, which binds to and inhibits TGF*β* [26]. Similar studies in transgenic mice overexpressing TGF*β*1 in a muscle-specific manner showed muscle wasting and endomysial fibrosis [27]. Finally, latent TGF*β*-binding protein 4 (LTBP4), which regulates the release and bioavailability of TGF*β* from the ECM, was recently shown to modulate fibrosis in mdx mice although its role in human DMD, if any, has not yet been established [28].

### 2.2. Crosstalk between TGF*β* and Other Growth Factors: CTGF and the Renin-Angiotensin System

Connective tissue growth factor (CTGF) is a nonstructural regulatory protein present in the ECM that has an important role in fibrosis. Skeletal muscle from DMD patients, dystrophic dogs, and mdx mice all show elevated levels of CTGF [29]. Fibrosis development can be reduced in mdx mice by systemic administration of a neutralizing antibody against CTGF [30]. Functionally, CTGF has the ability to reproduce or amplify the effects of TGF*β* on fibrosis. For example, it can induce collagen type 1, *α*5 integrin, and fibronectin much more potently than TGF*β* in fibroblasts [31]. Moreover, overexpression of CTGF in muscle of WT mice with an adenovirus vector carrying the CTGF cDNA sequence induces strong fibrosis [32]. Like for TGF*β*, decorin may also negatively regulate CTGF activity. Even if the exact mechanism is not known, it has been shown that the inhibitory action of decorin on CTGF activity depends on its capacity to directly bind to CTGF [33]. Interestingly, the same study showed that CTGF induces the expression of decorin, indicating a potential mechanism of autoregulation. Together, these results suggest a negative role for both TGF*β* and CTGF in muscular dystrophies by directly inducing fibrotic processes and inhibiting myogenesis [34]. A possible new role for CTGF in fibrosis was recently revealed by the observation that CTGF expression is decreased in dystrophic muscle when angiotensin-converting enzyme (ACE) is inhibited by enalapril [35]. ACE is a critical enzyme of the renin-angiotensin system (RAS) that regulates blood pressure. Angiotensinogen is mainly produced by the liver and then converted into angiotensin 1 (Ang 1) by the renin enzyme (also known as angiotensinogenase), which is secreted by the granular cells of the kidney. Ang 1 is subsequently converted into angiotensin 2 (Ang 2) by ACE (Figure 1). Angiotensin 2 produces its biological effect by signaling through the Ang 2 receptor type 1 (AT1) and the Ang 2 receptor type 2 (AT2). Over the past decade, various studies have showed that Ang 2 is involved in the development of fibrosis in different pathogenic conditions and organs [36]. Interestingly, it was shown that the RAS is activated in various muscular dystrophies such as DMD or congenital muscular dystrophy (CMD) [37] and that dystrophic muscle in humans has increased levels of ACE, which may explain the elevated CTGF levels, but this remains to be proved. Although the role of the RAS system in fibrosis is not completely clear, several studies have shown that Ang 2 also induces expression of fibrotic markers in myoblasts [38]. TGF*β* treatment of C2C12 myoblasts was also shown recently to significantly increase AT2 expression [39], therefore providing a putative link between TGF*β* signaling and CTGF expression. Interestingly, while Ang 2 seems to be profibrotic, recent data indicates that angiotensin 1–7 (Ang 1–7), an endogenous bioactive peptide derived from Ang 2, has opposite effect to Ang 2. Indeed, it has been shown that Ang 1–7 inhibits TGF*β*-Smad signaling* in vivo*, which in turn leads to the reduction of the profibrotic microRNA- (miR-) 21, decreasing greatly fibrosis development in dystrophic muscles of mdx mice [40].

## 3. ECM Remodeling by Matrix Proteases

In addition to molecules like TGF*β* and CTGF that promote ECM deposition, normal muscle repair also requires factors that regulate the proteolytic degradation of the ECM, for example, to facilitate satellite cell and myoblast migration, to remove the temporary scaffold laid down during regeneration, and to allow fiber growth. Clearly, if these factors become dysregulated, then ECM deposition, fibrosis, and loss of muscle function could ensue. We will only consider here recent developments to this group of molecules, which include serine proteases of the plasminogen activation (PA) system, the broad family of matrix metalloproteinases (MMPs), and their inhibitors, PAI-1 and the TIMPs, respectively (reviewed in [41, 42]). Several other publications cover more general aspects of their role in skeletal muscle and fibrosis [43]. The MMPs are a large family of zinc-dependent proteolytic enzymes that includes various collagenases (MMP-1, MMP-8, and MMP-13), gelatinases (MMP-2 and MMP-9), stromelysins (MMP-3, MMP-7, MMP-10, and MMP-11), membrane-type metalloproteinases (MMP-14, MMP-15, MMP-16, MMP-17, MMP-24, and MMP-25), and metalloelastase (MMP-12) [41]. MMPs facilitate inflammation and migration of myogenic, inflammatory, vascular, and fibroblastic cells to damaged tissue by degrading the ECM. MMPs are released from damaged muscle and infiltrating cells, but their function is controlled not only by simple expression and release, but also by the net MMP activity which reflects the relative amount of activated enzyme. MMP activation requires proteolytic cleavage of the inactive precursor, by either membrane-type matrix metalloproteinase 1 (MT1-MMP) [44] or plasmin, and cleavage of their corresponding inhibitors [45].

Regulation of MMPs is thus complicated and involves not only expression, but also activation. In addition, in some cases the activity of MMPs can amplify or synergize with serine proteases of the plasminogen activation system to mediate ECM remodeling during tissue repair. The main function of the PA system is to degrade fibrin and at its core is the zymogen plasminogen which is converted into the active enzyme, plasmin, by two plasminogen activators (PAs): tissue-type plasminogen activator (tPA) and urokinase-type plasminogen activator (uPA). There are several inhibitors of the PA system including plasminogen activator inhibitor 1 (PAI-1) and alpha2-antiplasmin that operate at the level of the PAs or plasmin, respectively [42]. The PA system plays an important role in muscular dystrophy. Studies in mdx mice have detected increased uPA expression in skeletal muscle, while genetic loss of uPA exacerbated dystrophy and impaired muscle function in mdx mice because uPA is required to prevent excessive fibrin deposition [46]. More recently it has been shown that the extracellular PAI-1/uPA balance is an important regulator of miR-21 biogenesis, controlling age-associated muscle fibrosis and disease severity in muscle dystrophy [17]. Genetic loss of PAI-1 in mdx mice was shown to promote muscle fibrosis through several mechanisms. Firstly, it altered collagen metabolism by increasing uPA-mediated proteolytic processing of TGF*β* in muscle fibroblasts. Secondly, it also activated miR-21 expression, which in turn inhibited PTEN and enhanced signaling via the AKT pathway, which controls cell proliferation and survival, thus endowing TGF*β* with a remarkable ability to promote cell proliferation. However, age-associated fibrogenesis and muscle deterioration in mdx mice, as well as advanced muscle fibrosis in young mdx mice lacking PAI-1, could be prevented by direct interference with miR-21 or the PAI-1 substrate uPA. Consistent with this, forced miR-21 overexpression in mdx muscle accelerated fibrosis and enhanced disease severity, whereas treatment of aged mdx mice with an inhibitor of miR-21 improved muscle homeostasis and reduced fibrosis. This is an important observation since fibrosis is normally considered irreversible at advanced ages. Further studies are needed to refine our understanding of the role of the PAI-1/miR-21 fibrogenic axis in skeletal muscle fibrosis and the disease course in DMD patients, but as the full role of the axis emerges, so too will new therapeutic targets. From all these findings, it seems that the pronounced fibrosis in human dystrophic muscles is at least partially related to an altered proteolytic activity in the dystrophic muscles due to imbalances in expression and activity of PA/MMP system components [47].

## 4. Inflammation-Driven Fibrosis

One of the hallmarks of DMD is the chronic cycles of myofiber necrosis and repair, which histologically manifest as a sustained infiltration of mononuclear cells in muscle tissue. Consequently, considerable efforts have gone into characterizing these inflammatory cells to define their functions and their differences between the various acute and chronic (transgenic/genetic) models of skeletal muscle injury. One of the first events after tissue damage is the invasion of inflammatory cells to the site of injury. In response to acute injury, the first cells infiltrating the muscle are mainly leukocytes belonging to the myeloid lineage, principally neutrophils and monocytes/macrophages ([48], reviewed in [49]). Any disruption in the coordinated initiation, progression, and resolution of inflammation can lead to persistent muscle damage and impairment of regeneration, which in many cases is also characterized by development of fibrosis as observed in the muscular dystrophies (reviewed in [18]).

Indeed, it is well known that fibrosis is preceded and influenced by inflammation in several pathologies. In the context of muscular dystrophy, previous studies showed that inflammatory macrophages are an important source of TGF*β* in the mdx diaphragm muscle, suggesting that they might contribute importantly to fibrosis development [14]. However, macrophage-depletion experiments or investigation of mouse models with impaired macrophage recruitment demonstrated a more complex role for macrophages in muscle repair and the mdx phenotype, being either deleterious [50] or beneficial for fibrosis development [46], depending on the model used for the study. Increasing evidence supports a key role of M2 “alternatively activated” macrophages in the development of fibrotic conditions, such as asthma and idiopathic lung fibrosis, as opposed to M1 “classically activated” proinflammatory macrophages [51]. These M2 macrophages are activated by Th2-derived cytokines, such as IL-13, and can be identified by specific cell surface markers such as CD206 (also known as mannose receptor). They also express high levels of TIMP-1 and the chitinase-like secretory lectins Ym1 and Ym2. Interestingly, TGF*β*, in conjunction with IL-13, may amplify the expression of arginase I in alternatively activated macrophages which is a key enzyme in the initiation of collagen synthesis by fibroblasts. Indeed arginase I produces the amino acid proline that is required for collagen synthesis [52]. Of note, the diaphragm of mdx mice contains CD206 positive macrophages expressing arginase I and TGF*β*, whose expression increases with age correlating with increased levels of IL-13 [16] suggesting the potential implication of Th2/alternative macrophage activation in dystrophic muscle fibrosis. Interestingly, muscles of DMD patients also show a correlation between the number of alternatively activated macrophages and collagen deposition [53]. Other studies have confirmed the presence of a subpopulation of alternatively activated macrophages, the M2c macrophages, a type of alternatively activated macrophage induced by the anti-inflammatory cytokine IL-10 and that express arginase, along with classically activated M1 macrophages within the dystrophic muscle of mdx mice [54]. Indeed, it has been shown that arginine metabolism by alternatively activated macrophages promotes cardiac and muscle fibrosis in mdx muscular dystrophy [55]. However, further investigations are required to elucidate the precise role of alternatively activated macrophages in fibrosis development in muscular dystrophies.

Although M1 macrophages are important for satellite cell proliferation (reviewed in [49]), they could potentially be profibrotic in pathogenic conditions by sustaining chronic inflammation. This is illustrated by the proinflammatory role of fibrinogen in muscular dystrophies. Fibrinogen accumulates in the dystrophic muscles of mdx mice and DMD patients [16, 46] and its genetic and pharmacological depletion in mdx mice can greatly reduce fibrosis development in the diaphragm [16]. Interestingly, fibrinogen modulates inflammation by signaling through the *α*_M_ *β*_2_ integrin (Mac-1), which is expressed on myeloid cells, and induces expression of proinflammatory cytokines and chemokines ([16] and reviewed in [56]), thereby potentially promoting muscle degeneration. Indeed, mice expressing a fibrinogen molecule mutated in its *α*_M_ *β*_2_-binding motif [16, 57] develop less fibrosis in the diaphragm concomitantly with a decreased infiltration of macrophages and decreased expression of proinflammatory cytokines, correlating with improved muscle regeneration.

Along with myeloid cells, lymphocytes have also been shown to play a role in muscular dystrophies. Among this family, a potential role of T helper (Th) and cytotoxic T cells (CTL) in fibrosis development has been raised recently. One of the most interesting animal models, to at least partially reveal the role of these cells in regeneration and fibrosis, is the scid/mdx mouse model. These mice are deficient in functional lymphocytes (both B and T cells), allowing the study of the function of these cells in the progression of the disease. Interestingly, scid/mdx mice develop less fibrosis in the diaphragm at one year of age, which correlates with a decrease in TGF*β* protein in the dystrophic muscle [58]. In addition, evidences for the pathogenic role of T cells in the progression of muscular dystrophies come from a study using another mouse model of immunodeficiency, the nu/nu/mdx mice, which lack only functional T cells, but not B cells [59], and showing that T cells contribute to fibrosis progression. Although these models support T cell function in muscle repair and muscular dystrophy, they unfortunately do not discriminate between the pathological effect of CTL and Th cells since both subpopulations are absent in scid/mdx and nu/nu/mdx mice. Using depleting antibodies against either the CD4 antigen (depletion of Th) or the CD8 antigen (depletion of CTL), it was shown that both cell types contribute to aggravation of the pathology in the context of mdx mice [60]. When analyzing the populations of leukocytes infiltrating the dystrophic muscle of mdx mice, Vetrone et al. characterized a subpopulation of T helper cells harboring a V*β*8.1/8.2 TCR (T-cell receptor) and expressing high levels of the ECM protein osteopontin ([61] and reviewed in [62]). Using mdx mice deficient in osteopontin, the authors showed that these mice have less fibrosis in the diaphragm muscle correlating with reduced infiltration of NKT cells and neutrophils. These data suggest that Th cells could mediate their pathogenic effects by controlling migration and/or survival of these inflammatory populations through the secretion of osteopontin.

Th cells can differentiate into different functional types, each of them producing a different profile of cytokines. Their role in fibrosis development in tissues other than skeletal muscle has been reviewed elsewhere [52, 63]. Unfortunately, since scid/mdx, nu/nu/mdx mice, or CD4 depleted mdx mice are totally deficient in Th cells, these models are unable to reveal the roles of their different polarizations (Th1, Th2, Th17, and Tregs) which probably play nonredundant and even opposite roles in the progression of fibrosis [64].

## 5. Fibroadipogenic Progenitors in Fibrosis

The existence of progenitor cells sharing characteristics of mesenchymal stem cells (MSCs) in skeletal muscle has been recently discovered [65, 66]. These cells were first named FAPs for fibro/adipogenic progenitors because of their capacity to enter adipose and fibroblast differentiation* in vitro* and* in vivo* [65], and were characterized as nonhematopoietic (CD45_−_), nonendothelial (CD31_−_), and nonsatellite cells (*α*7 integrin- or SM/C2.6- depending on the study), but they did express markers of progenitor cells such as CD34 or Sca-1 [65] or the fibroblast marker PDGFR*α* [66]. Although it is not assured that these two studies described exactly the same population of cells, it is likely that they are at least closely related or that they overlap. Controversies about their function in myogenesis and normal muscle regeneration exist [67, 68] and have been recently reviewed and discussed elsewhere [69], but it appears that these cells may have a pathogenic role in muscular dystrophies. Indeed, Uezumi et al. showed that fibrosis originates almost exclusively from PDGFR*α*+ progenitors in the dystrophic muscle of mdx mice [70].

Interestingly, a recent study characterized a subpopulation of cells expressing PDGFR*α*+, Sca1+, and gp38+, which also transiently expresses Adam12 upon acute injury [71]. Using a lineage tracing system, Adam12 expressing cells were shown to readily differentiate into myofibroblast* in vitro* and* in vivo*. These results suggest that the Adam12+ fraction might be a subpopulation of the FAPS described by Joe et al., which would be more committed to become bona fide fibroblasts. Indeed, depletion of the Adam12+ fraction of MSCs reduced the ECM accumulation induced by cardiotoxin injection. Although this study was performed in an acute model of injury, these findings are consistent with the fact that overexpression of Adam12 in mdx mice aggravates fibrosis [72] and suggests that this population of cells might be a major factor in fibrosis development within dystrophic muscle.

Unlike organs such as kidney or heart, where distinct cell types have been shown to contribute to fibrosis [73, 74], in skeletal muscle very little is known about the role of other cell lineages in fibrosis development and their potential contribution to the pool of fibroblasts. However, although MSCs are probably the main source of fibroblasts within the dystrophic muscle, additional studies suggest that other cells might also contribute to fibrosis in pathological contexts. For example, in aged mice, satellite cells tend to convert from a myogenic to a fibrogenic lineage in response to environmental cues, particularly in response to Wnt signaling [75]. More recently, Zordan and colleagues showed that macrophage infiltration after acute injury is required to sustain the proper differentiation of endothelial-derived progenitors. Indeed, depletion of macrophages after injury led to a transition of endothelial to mesenchymal cells [76].

## 6. Experimental Mouse Models of Skeletal Muscle Fibrosis

As it becomes increasingly accepted that fibrosis is a crucial component in the pathogenesis of DMD, the need for appropriate mouse models that reflect the human disease has become more and more urgent, since disease progression is less severe in mdx mice than in human patients. In the mdx mouse, fibrosis develops extensively and exclusively in the diaphragm muscle during adulthood [77], while in the more accessible limb muscles, it requires nearly two years for fibrosis to develop and it never reaches the severity of human disease [17]. This is despite limb muscles showing other histological features of the human disease, such as inflammatory infiltration, central nucleation, and both hypertrophied and small calibre fibers. Moreover, in the mdx mouse, several other clinical manifestations are mild in comparison to the human disease [78].

In order to exacerbate or hasten fibrosis, different genetic mouse models have been generated. Utrophin is a large sarcolemmal protein with many structural and functional features similar to dystrophin that is upregulated in muscle of DMD patients and may be able to partially compensate functionally for the loss of dystrophin. Transgenic mdx/utrn^+/−^ mice (mdx mice with haploinsufficiency of utrophin) were generated and were found to have increased inflammation of the hindlimb muscles at 3 and 6 months and in the diaphragm at 3 months only [79]. However, fibrosis was strong in the diaphragm at 6 months, but only mild in hindlimb muscle. Wishing to explore the question of whether differences in muscle stem cell (satellite cell) potential between mice and humans were responsible for the progressive DMD phenotype, Blau and collaborators generated dystrophic mice lacking telomerase activity by crossing C57Bl6 mdx mice with C57Bl6 mice heterozygous for the telomerase RNA component Terc (mTR) [80]. In addition to an enhanced fibrotic phenotype, these mice also had several other phenotypic characteristics of human DMD including profound loss of muscle force, endurance (performance on a treadmill), increased serum creatine kinase (CK) levels, accumulation of calcium deposits within the muscle tissues, and a shortened lifespan [81]. Another study explored differences between mice and humans by focusing on sialic acid composition of glycoproteins and glycolipids. Humans have an inactivating deletion in the CMAH (cytidine monophosphate-sialic acid hydroxylase) gene, which prevents biosynthesis of the sialic acid, N-glycolylneuraminic acid, from all human cells, although in mice this ability is not lost, and just as importantly, glycosylation has been shown to be a modifier of many disease states including mdx mice and *α*-sarcoglycan-deficient mice [82, 83]. Indeed, genetically engineered CMAH/mdx mice showed increased fibrosis, necrotic foci, and more central nucleation than mdx mice at 6 weeks of age, whereas by 8 months of age mice had lost ambulation and showed reduced force production. Studies by Ardite and colleages [17] showed that genetic loss of the uPA inhibitor PAI-1 in mdx dystrophic mice enhanced muscle fibrosis via several different mechanisms: firstly, it altered collagen metabolism by uPA-mediated proteolytic processing of TGF*β* in muscle fibroblasts; loss of PAI-1 also activated miR-21 expression (through a nongenomic TGF*β*-induced Smad activity), which in turn inhibited PTEN (phosphatase and tensin homologue) and enhanced AKT signaling, thereby endowing TGF*β* with a remarkable cell proliferation-promoting potential on fibroblasts.

Consistent with the notion that arginine metabolism by arginase-2 in M2 macrophages can drive fibrosis, and that the numbers of arginase-2-expressing M2 macrophages are elevated in the muscle and hearts of dystrophic mice, ablation of arginase-2 in mdx mice resulted in significantly less fibrosis in limb and diaphragm muscles [54]. Conversely, supplementation of young mdx mice with L-arginine promoted a more severe muscle fibrosis than mdx mice treated with D-arginine confirming a role for arginase in fibrosis and disease pathogenesis [54]. Of note, L-Arginine, but not D-arginine, is the natural substrate for nitric oxide synthase (NOS). Thus, caution needs to be taken since many DMD patients are often given arginine supplements, and more studies need to be undertaken to analyze its impact on disease.

A study by Fukada et al. showed that mice in the DBA/2 background exhibit a poor regeneration process after repeated injury. Furthermore, mdx mice in the DBA/2 background (D2-mdx) show severe loss of skeletal muscle weight and higher muscle weakness, while fibrosis and fat accumulation are greatly increased in comparison with mdx mice in the C57Bl10 background [84].

Nevertheless, neither of these mdxmouse models mimics precisely the pathophysiology of human dystrophic disease, and this is one of the reasons why it is difficult to study therapies to stop the progression of fibrosis in DMD.

In addition to genetic models, the drive to overcome our poor understanding of the molecular mechanisms underlying fibrogenesis in DMD has prompted the development of new experimental procedures that can be used to efficiently boost or advance fibrosis in young mdx mice in both hindlimbs without having to wait for its natural physiological onset.

Mechanical muscle injury by daily repeated micromultipunctures for 14 days has been demonstrated to trigger fibrotic lesions in mdx hindlimb muscles that consequently display a similar pattern to diaphragm muscles [85]. In this model, punctures are performed randomly on the whole tibialis anterior muscle surface. In contrast to toxin injection, which triggers whole muscle damage, the micropins induced several local myofiber injuries that, when repeated daily, trigger chronic injury. A more physiological method has been to use exercise training, which is known to exacerbate the process of muscle degeneration/regeneration by increasing fiber necrosis and amplifying inflammatory reactions [86]. After one month of training, fibrogenesis is induced in hindlimb muscles and is further aggravated by a prolonged exercise regime. Alternative and faster ways to trigger fibrosis in the limb muscle of mdx mice are based on surgical and chemical damage, such as laceration or denervation. Laceration consists in a deep cut across the muscle [87] which causes a delay in the healing process, while the denervation model involves severing the sciatic nerve, thus causing atrophy of the denervated myofibers [88]. These are two powerful methods for inducing sustained fibrosis, the disadvantage being the limited area of the muscle affected and thus available for sampling, or the number of muscles affected, respectively.

## 7. Treatment of Fibrosis and Clinical Perspectives for DMD

Fibrosis development is a consequence of the chronic degeneration and impaired regeneration of dystrophic muscle, which is itself caused by loss of the dystrophin gene. While the core aim of gene or cell therapy remains to replace the missing gene and thereby cure the disease at the roots by targeting the cause of muscle degeneration, preventing fibrosis progression should be considered an adjunct therapy for several reasons. Firstly, fibrosis development may negatively interfere with cell and gene therapies by reducing the amount of target tissue available for repair. Secondly, preventing fibrosis can also potentially improve quality of life and lifespan of dystrophic patients on its own. Several pharmacological treatments targeting fibrotic cells or molecules are currently being tested and some are showing promising effects in human and animal models [89].

One of the signaling pathways involved in fibrosis development is the RAS system (as discussed above). Several components of this system have been used as targets to decrease dystrophic muscle fibrosis in animal models. Administration of the angiotensin 2 type 1 receptor antagonist losartan, which is commonly used to treat high blood pressure, has been shown to improve muscle strength and ameliorate fibrosis in dy(2J)/dy(2J) mice with laminin-*α*2-deficient congenital muscular dystrophy [90]. Other studies have also shown improved muscle function and diminished fibrosis in mice following losartan treatment associated with cardiotoxin injury and hindlimb immobilization-induced sarcopenia [91], decreased muscle fibrosis after laceration [92], and decreased cardiac fibrosis in mdx mice [93].

Other molecules target the RAS by inhibiting ACE. For example, lisinopril is an ACE-inhibitor that has been shown to preserve cardiac and skeletal muscle integrity in mdx mice [94]. Indeed, because of the positive preclinical effects shown for losartan and lisinopril, a recent double-blind randomized clinical trial was commenced to compare lisinopril versus losartan for the treatment of cardiomyopathy in human DMD patients [95]. Both drugs have already been shown to be effective for the treatment of dilated cardiomyopathy. Similarly to lisinopril, administration of the ACE inhibitor enalapril to mdx mice was shown to decrease skeletal muscle fibrosis [35]. Interestingly, treatment of mdx mice with the peptide angiotensin-1–7 (Ang 1–7) had the opposite effect to that of angiotensin 2, in that it improved muscle fibrosis by inhibiting TGF*β* signaling and concomitantly decreasing the number of fibroblasts [40]. Taken together, these findings suggest that targeting the RAS may be a promising way to delay fibrosis progression in DMD.

Targeting profibrotic growth factors or cytokines to slowdown fibrosis development has also showed promising results. Administration of the antibody FG-3019, which neutralizes CTGF, or the administration of an anti-TGF*β* neutralizing antibody improves the phenotype of mdx mice by delaying fibrosis development [96]. However, as TGF*β* and CTGF are pleiotropic, molecules, targeting them often induces undesired side effects such as increasing the amount of proinflammatory CD4+ T cells infiltrating the muscle [96].

Imatinib is an inhibitor of tyrosine kinase receptors, including PDGFR*α*, which was used originally for human cancer therapy. It has been shown that its administration is beneficial for muscular dystrophy in mdx mice [97]. Interestingly, imatinib appears to target specifically mesenchymal progenitors by inhibiting both their proliferation and expression of fibrosis markers* in vitro* [98]. Other molecules, known to have anti-inflammatory effects, have also shown beneficial effects by delaying fibrosis progression in mdx muscle, including halofuginone, a synthetic halogenated derivative of the naturally occurring molecule febrifugine [99]. Halofuginone was shown to greatly improve muscle histopathology and fibrosis in a model of dysferlin deficient mice [100]. Interestingly, halofuginone is known to inhibit specifically Th17 cell differentiation [101], suggesting that this molecule might act by modulating inflammation in the dystrophic muscle. Indeed, a recently concluded clinical trial (reference NCT01847573, http://clinicaltrials.gov/show/NCT01847573) has assessed the safety and tolerability of halofuginone in DMD patients, but no data on efficacy will be available for some time. However, despite several drugs showing promise in animal models, few have currently progressed to clinical trials in DMD patients.

## 8. Concluding Remarks and Perspectives

Fibrosis is an excessive deposition of ECM components that sometimes occurs as a result of dysregulated or chronic damage and repair processes. Human DMD patients have characteristic signs of muscle necrosis and repair with persistent fibrosis in muscles at young age, which play a significant role in the progressive nature of the disease and the reduced life expectancy. However, the mdx mouse model of DMD does not normally develop fibrosis extensively in the limbs, particularly at young age, and has several other differences from the human disease. Here we have reviewed some of the recent literature that has tried to bridge this species difference in order to develop better models to study the human condition, to improve existing treatments, and to open the door for new ones. Recent animal models have been shown to be better tools for unraveling the roles of new cell mediators in repair and fibrosis, including inflammatory and mesenchymal cell subpopulations, but the identity of a real ECM-producing cell in skeletal muscle remains elusive. Moreover, cell sorting techniques in mice are often complicated to reproduce in human patients due to differences in cell surface markers and the unavailability of sufficient sample material to extract significant numbers of cells. Advances have been made in the identification of new growth factors and cytokines and their downstream signaling components, which are also important targets for ameliorating fibrosis. As well as new advances in targeting TGF*β* with antibodies, the more recent implication of the RAS system in fibrosis may also prove important mechanisms as there are already many approved drugs on the market that are able to modulate this system and potentially produce beneficial effects. However, despite all the promises shown in treating mice, there have been few clinical trials to date. While this is frustrating to patients and families, there is also a need for caution in advancing too quickly since there are still many unknowns and many clear differences in muscle pathology and fibrosis development between human DMD patients and mdx mice that may respond unexpectedly to treatment. Nonetheless, these differences are growing smaller as new animal models and new molecular tools are developed.
